# Ultrastructure of noise-induced cochlear synaptopathy

**DOI:** 10.1038/s41598-023-46859-6

**Published:** 2023-11-09

**Authors:** Daniel J. Moverman, Leslie D. Liberman, Stephan Kraemer, Gabriel Corfas, M. Charles Liberman

**Affiliations:** 1 Eaton-Peabody Laboratories, Massachusetts Eye and Ear, 243 Charles St., Boston, MA 02114-3096 USA; 2 Center for Nanoscale Systems, Harvard College, Cambridge, MA 02138 USA; 3 Department of Otolaryngology-Head and Neck Surgery, Kresge Hearing Research Institute, University of Michigan, Ann Arbor, MI USA; 4 Department of Otolaryngology-Head and Neck Surgery, Harvard Medical School, Boston, MA 02115 USA

**Keywords:** Auditory system, Diseases of the nervous system, Nervous system

## Abstract

Acoustic overexposure can eliminate synapses between inner hair cells (IHCs) and auditory nerve fibers (ANFs), even if hair-cell function recovers. This synaptopathy has been extensively studied by confocal microscopy, however, understanding the nature and sequence of damage requires ultrastructural analysis. Here, we used focused ion-beam scanning electron microscopy to mill, image, segment and reconstruct ANF terminals in mice, 1 day and 1 week after synaptopathic exposure (8–16 kHz, 98 dB SPL). At both survivals, ANF terminals were normal in number, but 62% and 53%, respectively, lacked normal synaptic specializations. Most non-synapsing fibers (57% and 48% at 1 day and 1 week) remained in contact with an IHC and contained healthy-looking organelles. ANFs showed a transient increase in mitochondrial content (51%) and efferent innervation (34%) at 1 day. Fibers maintaining synaptic connections showed hypertrophy of pre-synaptic ribbons at both 1 day and 1 week. Non-synaptic fibers were lower in mitochondrial content and typically on the modiolar side of the IHC, where ANFs with high-thresholds and low spontaneous rates are normally found. Even 1 week post-exposure, many ANF terminals remained in IHC contact despite loss of synaptic specializations, thus, regeneration efforts at early post-exposure times should concentrate on synaptogenesis rather than neurite extension.

## Introduction

In both noise-induced and age-related hearing loss, the synaptic connections between hair cells and auditory nerve fibers (ANFs) are among the most vulnerable elements in the inner ear. For example, after an acoustic overexposure that causes a large, but fully reversible, threshold elevation there can be a permanent loss of up to 50% of the ANF synapses over large regions of the cochlear spiral^1,2^. This persistent cochlear neural degeneration has minimal effect on overall threshold sensitivity, in part, because the loss is biased towards the normal subgroup of fibers with high thresholds and low spontaneous rates (SRs), at least in noise-exposed guinea pigs^3^ and in aging gerbils^4^, although an SR-based selectivity is not so clear in mice^5^. These low-SR fibers are thought to be particularly important for stimulus coding in a noisy environment^6^, thus, problems understanding speech in noise, the most common complaint of people with sensorineural hearing loss, may arise predominately from this type of primary cochlear neurodegeneration.

Although cochlear synaptopathy has been well-studied in a number of species, and after a variety of types of noise exposure^7^, all of the quantitative histopathological analysis of this phenomenon has been done at the light-microscopic level, using immunohistochemistry to visualize key elements of the pre- and post-synaptic machinery (e.g.^1–3,8,9^.). In the normal ear, each ANF has a bipolar cell body in the spiral ganglion and sends a myelinated central axon to the brainstem, and a single, myelinated peripheral axon to the organ of Corti^10^. As they enter the organ of Corti, the peripheral axons lose their myelin and each typically extends a single peripheral terminal to make a synaptic contact with a single inner hair cell (IHC)^11^. On the IHC side of this synaptic connection, ultrastructural images show an electron-dense, pre-synaptic ribbon closely apposed to the IHC membrane and surrounded by a halo of vesicles^12–15^; on the post-synaptic side, the membrane of the ANF terminal appears thickened due to the glutamate receptors and other proteins clustered at the synaptic active zone^13^. Thus, immunostaining for a ribbon protein (CtBP2) and a glutamate receptor subtype (usually GluA2) produces confocal images with closely apposed puncta that can be counted to reveal the number of synapses in normal and noise-exposed ears^16,17^.

Confocal studies of the post-exposure dynamics of noise-induced synaptopathy show a dramatic reduction in the number of pre- and post-synaptic puncta immediately at the end of the synaptopathic noise exposure, and, at least in CBA/CaJ mice, little to no recovery at longer survival times^1,2,18^. Since the immunohistochemical studies only show the level of protein expression, it is not clear to what extent the ANF terminals degenerate and retract, or alternatively internalize glutamate receptors at the synapse, as has also been suggested^19^. Ultrastructural studies of the acute effects of noise have shown what appear to be the grossly swollen ANF terminals, with broken membranes, opposite relatively normal-looking pre-synaptic ribbons in intact hair cells^20,21^. These ultrastructural observations suggest that the terminals rupture and retract from the IHC soon after exposure. Indeed, this type of terminal retraction has seen in vitro when neonatal cochlear explants are treated with glutamate agonists^22^. However, the long-term synaptopathic outcomes of the exposures used in prior ultrastructural studies are not known, and, at the light-microscopic level, it is difficult to count the unmyelinated terminals of ANFs in adult cochleas, because of the complexity of the neuropil under the IHCs. Thus, the time course of retraction of the ANF peripheral dendrites in the classic synaptopathy model is poorly characterized, although it is clear that the ultimate death of the cell body requires months to years^1^.

Understanding the temporal dynamics of the ultrastructural synaptic changes is critical, because attempts to reverse the noise-induced loss of synapses by raising intracochlear levels of neurotrophins have been successful, but only when the trauma-treatment interval is less than or equal to 1 day^23^, while treatments even as short as 1 week post exposure have been unsuccessful (Liberman, unpublished). Here, we harness the power of FIB-SEM serial-section ultrastructure and machine-learning driven auto-segmentation to study the ultrastructure of synaptophy in the best-studied model system, the noise-exposed CBA/CaJ mouse. We find that, at both 1 day and 1 week post-exposure, most ANF terminals remain intact and in contact with the IHC, however, they lack all conventional synaptic specializations.

## Methods

### A. Animals and groups

Male CBA/CaJ mice were obtained from our supplier at 6–7 weeks of age and allowed to acclimate for 1 week before any experimental manipulation. Noise exposures were carried out at 8 weeks of age, with the animals awake and unrestrained in small cages placed on a rotating platform within a uniform sound field (± 1 dB). Two unexposed animals served as controls: one was 8 weeks at the time of tissue harvest and the other was 13 weeks. Exposures were to an octave-band noise (8–16 kHz) at 98 dB SPL for 2 h, then animals were allowed to survive for 1 day or 1 week post exposure before tissue harvest. Initial tissue fixation was accomplished by intravascular perfusion of a mixed aldehyde solution (2.5% glutaraldehyde, 1.25% paraformaldehyde) in cacodylate buffer, followed by intralabyrinthine perfusion and post-fixation in the same solution for 10 h at 4 °C, and decalcification in cacodylate-buffered 5% EDTA for 2 days at 4 °C. All procedures were approved by the Institutional Animal Care and Use Committee of the Mass Eye and Ear. All experiments were performed in accordance with the relevant guidelines and regulations and are reported in accordance with the ARRIVE guidelines^24^.

### B. Histological preparation

#### 1. En bloc staining

Decalcified cochleae were microdissected into 6 pieces, each containing a fractional turn of the osseous spiral lamina with the organ of Corti and spiral ligament attached. Subsequent *en bloc* staining with heavy metals used an approach closely modeled after that from a prior report^12^. In brief, the pieces were washed in 0.15M cacodylate (pH 7.4; 2 × 30 min) and sequentially immersed in the following solutions (all buffered with 0.15M cacodylate): 2% OsO_4_, 2.5% ferrocyanide, and again in 2% OsO_4_ at room temperature for 2, 2, and 1.5 h, respectively, without intermediate washes. After sequential cacodylate and water washes (3 × 10 min each), cochleae were incubated at 40 °C in a 1% aqueous solution of thiocarbohydrazide for 45 min, washed in water 6 × 10 min, and then incubated in unbuffered aqueous 2% OsO_4_ for 2 h. After an overnight water wash the pieces were transferred to lead aspartate (0.03 M, pH 5.0) at 50ºC for 2 h, followed by a final water wash (6 × 10 min).

#### 2. Cochlear mapping

During the *en bloc* staining procedure, while the pieces were rinsing prior to the thiocarbohydrazide step (when the tissue becomes opaque and cellular detail is lost), the six microdissected pieces were arranged in order and photographed through a dissecting microscope. A cochlear frequency map was produced from this micrograph, by tracing an arc along the heads of the pillar cells in each piece, using a custom ImageJ plugin developed for use in light-microscopic studies of cochlear synaptopathy (https://meeeplfiles.partners.org/Measure_line.class).

#### 3. Plastic embedding

After the en bloc staining, cochlear segments were dehydrated and plastic-embedded in a sequence modified from a prior report^25^. Pieces were advanced through an ascending ethanol series (50%, 70%, 90%, 30 min each at 4 °C, and 100% for 4 × 15 min at room temp), followed by propylene oxide (3 × 20 min). Pieces were then infiltrated with a mixture of plastic resins (7.5 ml Araldite 502, 12.5 ml Embed-812, 27.5 ml DDSA, from EMS) thinned with propylene oxide in the following ratios: propylene oxide:resin at 3:1 for 4 h, 1:1 overnight, and 1:3 for 8 h. Tissue pieces were then incubated overnight in a degassed mixture of resin and accelerator (200 μl DMP-30 plus 10 ml resin), transferred the next day to fresh resin plus accelerator in flat molds (EMS 70902), positioned with organ of Corti in a surface view, and polymerized at 60 ºC for 48 h.

#### 4. Final microdissection and FIB-SEM sample preparation

After polymerization, the flat-embedded cochlear piece containing the desired frequency region (32 kHz for this study) was removed from the mold, and the plastic above and below the organ of Corti was ground away with sanding discs to produce a thin wafer amenable to further microdissection with razor blades. Using the micrographs from the mapping procedure for orientation, a small wedge of the organ of Corti containing ~ 100 mm of the cochlear spiral centered exactly on the 32 kHz region was cut out and glued on one cut side to the tapered end of a BEEM-capsule-molded plastic stub (organ of Corti perpendicular to the face of the stub). One-micron plastic sections were cut on an ultramicrotome to check the orientation and to produce a flat face for the subsequent FIB-SEM procedure.

#### 5. FIB-SEM milling and imaging

Each plastic-embedded cochlear block was positioned in the Zeiss Cross Beam 540 so as to mill and image in a plane parallel to the basilar membrane. The final image stacks were cubes roughly 20 mm on each side, imaged with 5 nm resolution in x and y and with 20 nm section thickness. At that size and resolution, each image stack required 18–24 h of imaging time. After acquisition, images were aligned and denoised using custom MATLAB scripts.

### C. Auto-segmentation and other image analysis

#### Segmentation of afferent fibers

Auditory-nerve fibers were semi-automatically segmented with Dragonfly using the Active Contour plugin. After manually outlining a fiber in every 10–15 slices, a “Generate from Selected Paths” command automatically generates an ROI from the column of 3D rings. For a handful of fibers minor post-processing was required to match the outline to the fiber borders.

#### Quantification of efferent innervation

To quantify the efferent innervation of ANFs, the sections in which synapses were present were identified (see Results for description of the criteria). Then, the surface area of each synaptic contact was measured by manually tracing the region of apposed membranes in each section in Dragonfly using the Path Tool under 2D paint mode with a brush radius of 1 pixel. All the serial tracings for each contiguous synaptic zone were combined into a single ROI and the surface area of each synaptic plaque was estimated.

#### Mitochondrion auto-segmentation

Mitochondria were auto-segmented in Dragonfly using the Artificial Intelligence Deep Learning Tool. To create training data, roughly 1600 mitochondria were manually labelled in our first image stack. We trained the model exclusively on mitochondria within ANFs, because mitochondrial morphology differs according to cell type. In subsequent stacks, much less manual labelling was required (e.g. 600 in the second stack, then 200 in the third). From within the Dragonfly library of models, the encoder-decoder network of U-Net++ was chosen for this semantic segmentation task based on prior reports demonstrating its higher Intersection over Union in medical imaging^26^ and its attainment of a lower validation loss (i.e. the success of the model in analyzing the validation set) compared to U-Net^27^. For the structural parameters: the class count was 2, the depth level was set to 6, the initial filter count was set to 64, and the input dimension was 2.5D with its input slice count set at 7 (and slice 4 serving as the reference slice). This one model was trained and applied sequentially to all of our image stacks, where we measured its success by monitoring for the validation loss, which reached a final value within the range of 0.0050–0.0065 for each stack. The training loss was also monitored. When the model was applied to each stack, the final training loss was never lower than the final validation loss, confirming that the model was generalizable and not overfitting to the stacks. Furthermore, the software saves data from an epoch if and only if its validation loss decreases from the previously recorded low, and it terminates the run after “n” epochs without any decrease in validation loss. If a lower validation loss was desirable, additional mitochondria were manually labelled before re-running the model. The ultimate proof of the accuracy of the automatic segmentation was the careful visual evaluation of the correspondence between the machine-generated masks and the underlying morphology in every section through every ANF analyzed in the present study.

### D. Statistics

Statistical testing was performed in Prism 10.0.3, using the Kruskal–Wallis non-parametric test with Dunn’s multiple comparisons test when comparing more than two groups, and the Mann–Whitney non-parametric test when comparing two groups. P values for inter-group differences that were statistically significant (P < 0.05) are shown in the Figures.

## Results

### A. The synaptopathy model

**Figure 1:**
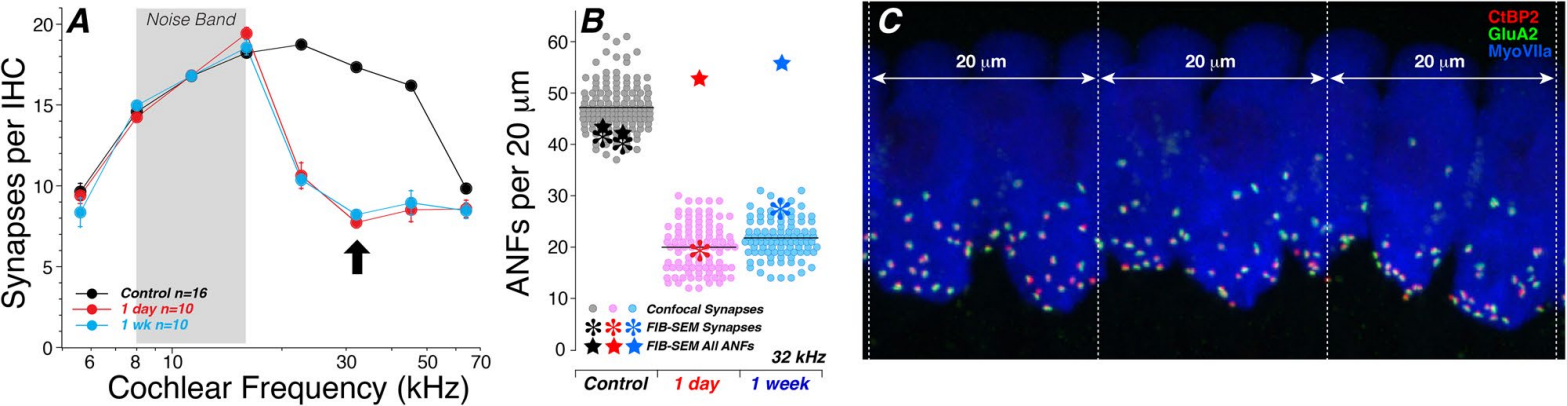
The noise exposure used here creates a reproducible and uniform pattern of synaptopathy, as demonstrated in prior confocal studies. (**A**) Counts of synapses per IHC at 8 cochlear locations, in control mice (n = 16) vs. those examined 1 day (n = 10) or 1 week (n = 10) after synaptopathic exposure in a prior study^2^. Mean values (± SEMs) were extracted from sets of confocal z stacks, each spanning 200 mm (~ 20 IHCs). Arrow indicates the 32 kHz region. (**B**) From the 32-kHz region in this prior confocal data, we extracted the number of synapses per 20 mm, i.e. the approximate span of the FIB-SEM stacks in the present study (also at 32 kHz). Each filled circle represents a different 20 mm sample. The asterisks show the synaptic counts extracted from each of the four FIB-SEM stacks in the present study, and stars indicate the total number of ANF terminals in each stack: both numbers were adjusted in proportion to the extent to which the stack span differed from exactly 20 mm. (**C**) A maximum projection of part of a confocal z-stack of the type used to extract the values in (**A**,**B**). Synapses appear as closely juxtaposed red and green puncta.

The model of synaptopathy used here is one we have studied extensively at the light-microscopic level^1,2,5,23^. It is designed to produce only a transient elevation of cochlear thresholds, but massive and permanent loss of the synapses between auditory nerve fibers (ANFs) and cochlear sensory cells, despite no loss of the hair cells themselves. After this exposure to an 8–16 kHz noise band at 98 dB SPL for 2 h, the loss of synapses, as seen via immunostaining of pre-synaptic ribbons and post-synaptic glutamate receptors (Fig. 1C), peaks at the 32 kHz region, where only about 50% remain, when viewed either at 1 day or 1 week post exposure (Fig. 1A).

In prior studies, the sampling power of confocal microscopy allowed us to extract synaptic counts from literally hundreds of hair cells at each of multiple cochlear regions, from many animals in each experimental group. In the present study, the labor-intensive nature of ultrastructural investigation limits us to a much smaller sample, i.e. one 20-μm cochlear span (~ 2 inner hair cells wide) from each of four ears. Thus, the consistency of the synaptopathic outcome in randomly selected 20 mm samples is key to evaluating the reliability of the present results. To this end, we re-analyzed data from confocal image stacks taken at the 32 kHz region, the heart of the synaptopathic region, extracting synaptic counts from contiguous 20 mm samples from the relevant z-stacks in prior studies, As shown in Fig. 1B, the synaptic counts per 20 mm from the confocal data are non-overlapping between control and exposed ears at either survival time. Furthermore, the synaptic counts from the present study (see below) all fit well within the prior light-microscopic data.

### B. Synaptic analysis

**Figure 2:**
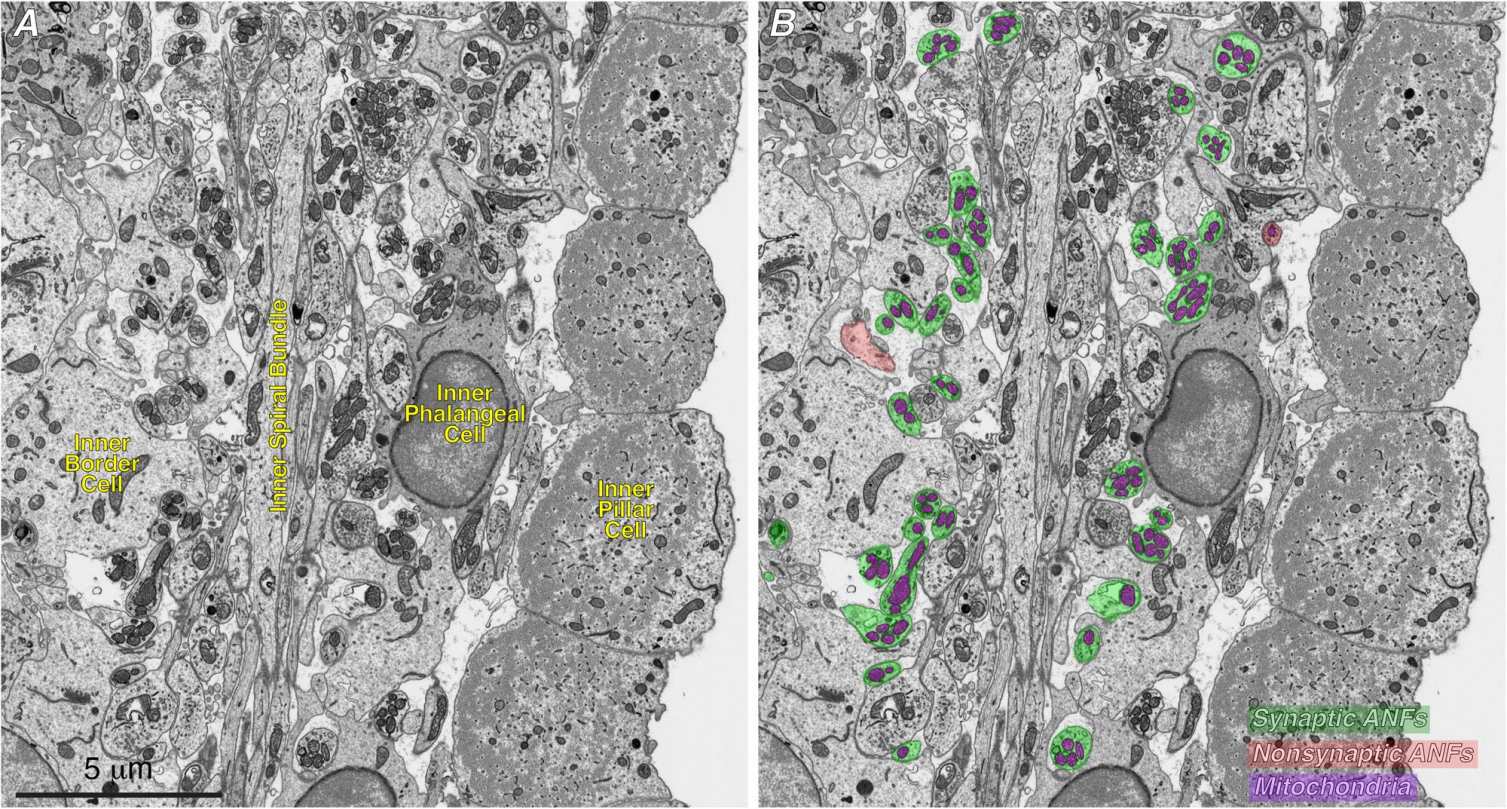
We used machine learning to automate much of the ultrastructural analysis. (**A**) Representative low-power micrograph of one slice of a control FIB-SEM stack from the region below the IHCs. (**B**) The same slice as in (**A**), with masks superimposed on all the mitochondria within ANFs, as found by the auto-segmentation routine, as well as masks superimposed on all the afferent fibers, color coded as to whether or not they ultimately formed a synapse with the IHC.

In the present study, we analyzed four FIB-SEM blocks, all from the 32 kHz region, two from control ears, one from an exposed ear at 1-day post exposure and one from an exposed ear at 1-week post exposure. In each case, we segmented the inner hair cells (IHCs), each ANF from near the habenula, where it enters the organ of Corti, to its terminus at or near the IHC, and all of the mitochondria they contained. One exemplar section is shown in Fig. 2, with and without the segmentation masks for mitochondria and ANFs. As described in Methods, the autosegmentation model for mitochondria was trained only on contours from within ANFs. As seen in Fig. 2B, the model accurately captured all the ANF mitochondria.

In the two control ears, a total of 75 ANFs were reconstructed, of which 71 formed synapses with an IHC and 4 did not. Of the non-synaptic fibers, two touched an IHC while the other two ended blindly without doing so. Of all control fibers from two separate blocks, 73/75 were unbranched, forming a single synapse via a single terminal swelling. One fiber branched to make synaptic contact with two adjacent IHCs, the other made two synapses with the same IHC.

**Figure 3:**
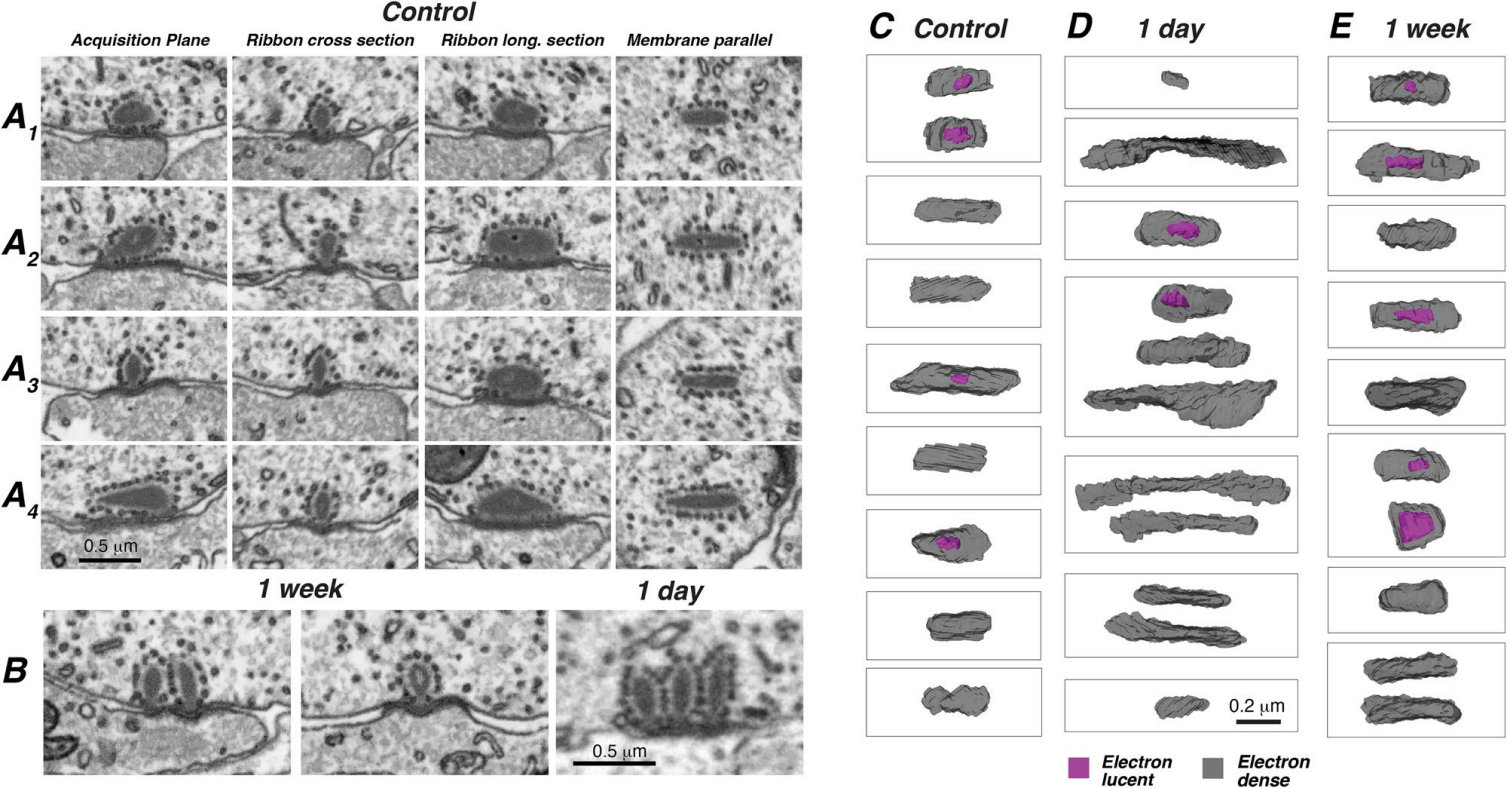
Pre-synaptic ribbons hypertrophy and duplicate after synaptopathic noise. (**A**_**1**_**–A**_**4**_) The apparent heterogeneity of ribbon morphology disappears when each synapse is virtually re-sectioned in a ribbon-centric plane, as enabled by the isotropism of FIB-SEM image stacks. Four examples from a control ear appear different from each other when viewed in the *acquisition plane*. The same four synapses look similar to each other when viewed either as either (2nd column)—*ribbon cross-section*, i.e. perpendicular to the subjacent IHC membrane and to the long axis of the ribbon; (3rd column)—*ribbon longitudinal section*, i.e. perpendicular to the membrane and parallel to the long axis of the ribbon; or (4th column)—*membrane parallel* to the adjacent IHC and through the middle of ribbon thickness. (**B**) Double and triple ribbons are more common in noise-exposed ears, at either 1-week or 1-day survival as indicated. (**C**–**E**) The post-exposure hetererogeneity of ribbon length and number is illustrated by this sampler of ribbon morphologies from control, 1-day and 1-week cases, respectively. For each survival time, the sample include the longest and shortest examples from the group. Each box outlines the ribbon(s) at a different ANF synapse. Each image is a 3D reconstruction of the ribbon volume, viewed perpendicular to the plane of subjacent IHC membrane, i.e. in the same orientation as the *membrane parallel* images from panels (**A**_**1**_**–A**_**4**_). Each surface is rendered with enough transparency to see the hollow core (purple) when present. When multiple ribbons are present, their relative orientations are maintained.

The morphology of the presynaptic ribbons in control ears can appear heterogeneous (Fig. 3A1–A4 1st* column*); however, when virtually re-sectioned in planes customized to the synapse’s orientation around the roughly hemispherical basal pole of the IHC, most of the heterogeneity disappears (Fig. 3A1–A4 columns 2–4). In the cross-sectional views (2nd column), all the classic features of the ribbon synapse are seen: an electron-dense presynaptic ribbon in the IHC, surrounded by a halo of vesicles, and closely apposed to a region of thickened post-synaptic membrane^13^. In three dimensions, each ribbon is a flattened cylinder, significantly longer than its diameter. Each is oriented with its long axis parallel to the IHC membrane, and some appear to have an electron-lucent core (Fig. 3C–E). In control ears, almost all synapses had a single ribbon at the active zone (69/71), while only two (3%) had a double ribbon.

**Figure 4:**
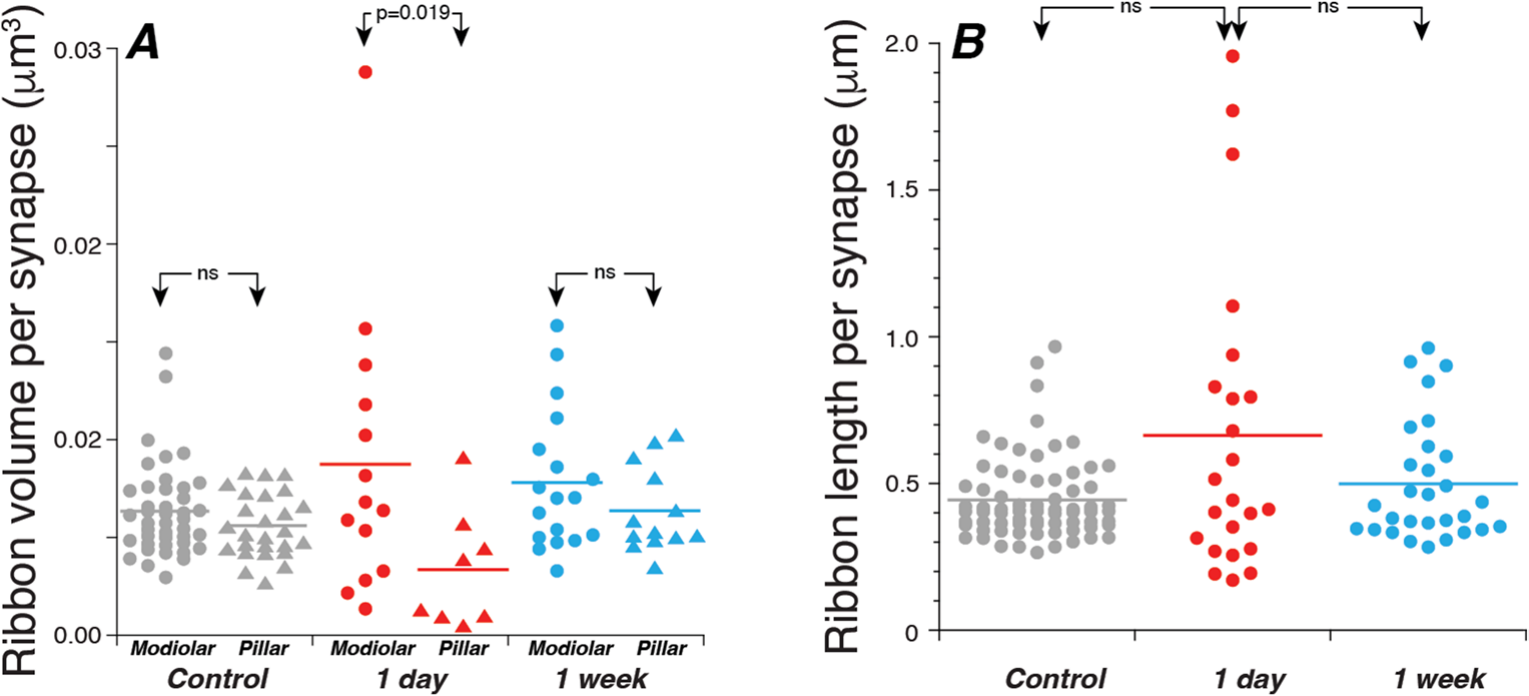
Total ribbon volumes (**A**) and ribbon lengths (**B**) for all the synapses in the present study. When there was more than one ribbon at a synapse, the volumes (and lengths) were added together for this plot, since they are too close to be resolvable with light microscopy. Horizontal lines indicate the mean of each distribution. Data from both control image stacks are combined here in both panels. Each of the four volumes analyzed spans ~ 20 mm of cochlear length, as described in Methods. P values for comparisons across survival times are not indicated in (**A**).

In exposed ears, we reconstructed 59 ANFs at each of the two survival times evaluated. Multiple-ribbon synapses were much more common in these exposed ears (Fig. 3B,D,E). In the 1-day case, 7 of 22 synapses (32%) had multiple ribbons (5 doubles and two triples), and in the 1-week case, 9/28 synapses (32%) had multiple ribbons (8 doubles and 1 triple). Some examples are shown in Fig. 3B. In addition to the multiplication of ribbons per synapse, ribbon shape at 1 day post-exposure was more heterogenous: some were exceptionally long, while others were exceptionally short compared to those seen in controls (Figs. 3C–E and 4B). Although the multiplication of ribbons was apparently long lasting (3% in controls vs. 32% at both 1 day and 1 week), the heterogeneity of ribbon shape had largely disappeared by 1 week post exposure (Fig. 4B).

In normal ears, a spatial gradient of ribbon sizes has been seen via confocal microscopy, i.e. larger on the modiolar side than the pillar side^17^. Our 3D reconstructions of ribbon volume showed the same trend in both control and exposed ears, however the modiolar vs. pillar group difference only reached statistical significance for the 1-day stack (Fig. 4). For the data in Fig. 4, we separated modiolar from pillar with a single plane for each image stack, as in our prior confocal analyses^2,17,18^. The conclusions were identical if the modiolar-pillar distinction was defined based on the position around each IHC, as has been done in prior ultrastructural studies^13,28^.

**Figure 5:**
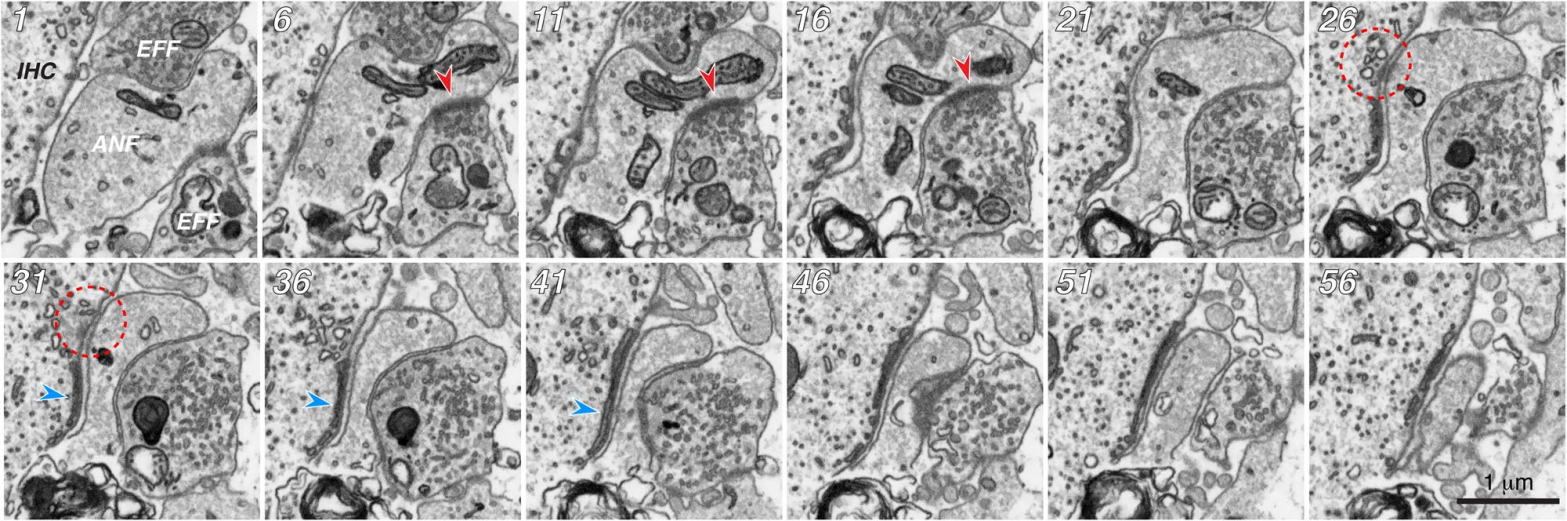
Many ANFs in the exposed ears contact the IHC but form no typical synaptic specializations. These 12 images span the entire region of IHC contact for one ANF from the 1-week survival case. Every 5th section is shown, as indicated by the numbers in the upper left: increasing numbers are closer to the top of the IHC. Dashed red circle indicates the possible prior location of the synaptic ribbon. Blue arrowheads in 31, 36 and 41 point to the prominent juxta-membranous cisterna present in the IHC. Contacts with two efferent terminals are visible, but only the regions indicated with red arrowheads from 6 to 16 were considered synaptic.

The most striking abnormality in the exposed ears was the large number of non-synaptic ANFs, i.e. fibers that failed to form any of the classic pre- or post-synaptic specializations, despite the fact that many of these non-synaptic terminals came in intimate contact with the IHC. One of these non-synaptic IHC contacts is illustrated in Fig. 5, where we show every 5^th^ slice through the entire contact zone. Although there is a complex web of juxta-membranous cisternae within the IHC that seems localized to the contact zone, there is no pre-synaptic ribbon, no cluster of synaptic vesicles and no obvious zone of post-synaptic membrane specialization. It’s possible that the cloud of large irregular vesicles and nearby diffuse electron density represents the former site of the pre-synaptic ribbon (red dashed circles in panels 26 and 31). In the 1-day stack, 37/59 ANFs (62%) were non-synaptic (21 of which (57%) were in intimate contact with an IHC), and in the 1-week stack, 31/59 (53%) were non-synaptic (15 of which (48%) were in intimate contact with an IHC). Among the non-synaptic fibers no longer in contact with the IHCs, most had not retracted very far: the average distance to the nearest IHC was 2.1 mm for both the 1-day and the 1-week survivals.

**Figure 6:**
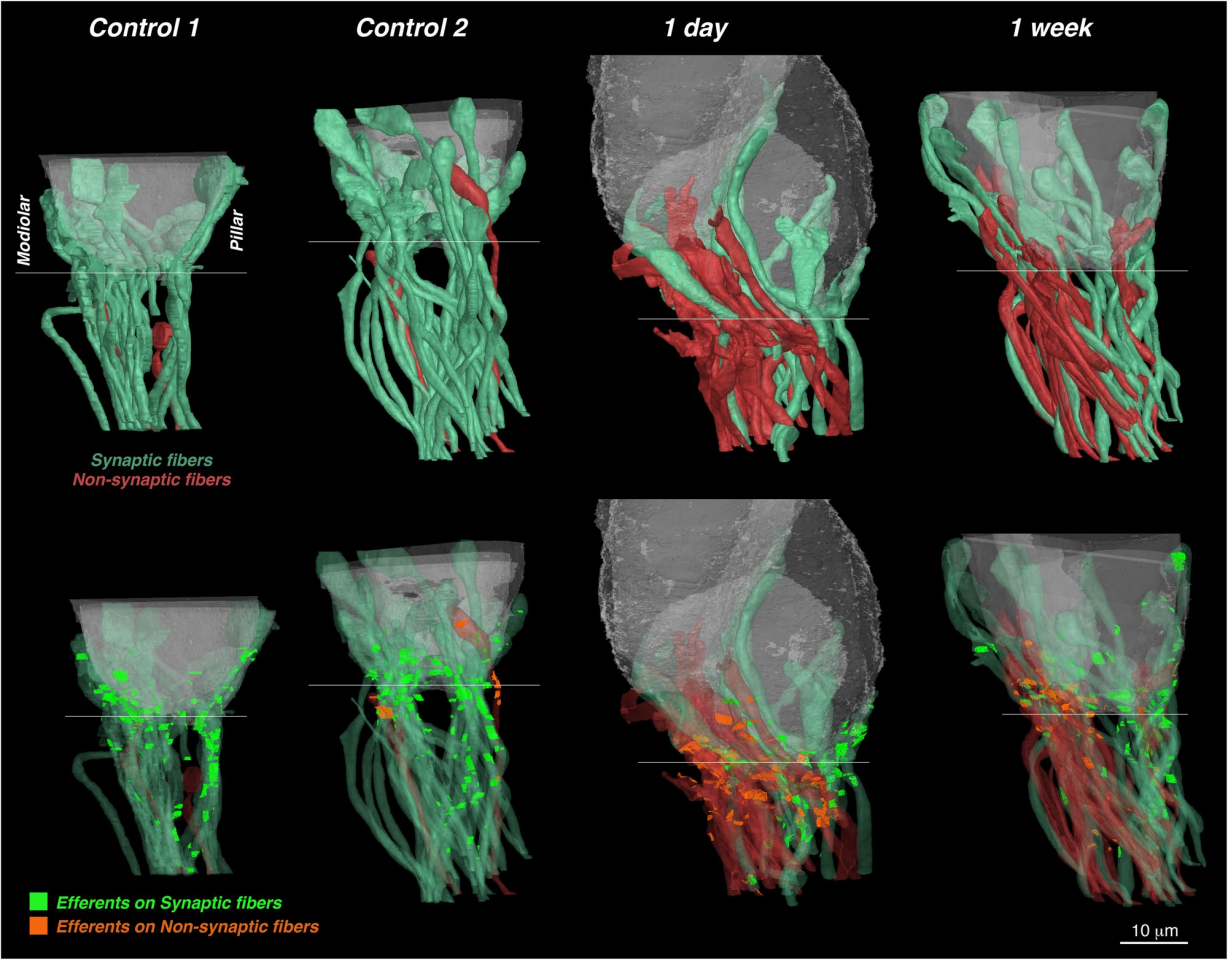
Semi-transparent 3D renderings of the IHCs, ANFs and efferent synapses for the four FIB-SEM stacks, each oriented with the modiolar faces of the IHCs to the left and the pillar faces to the right. The top row of images emphasizes the differences between ANFs making synaptic contacts (green) vs. those not (red). The bottom row shows the locations of the efferent contacts, colorized according to which type of ANF they synapse with. To generate the efferent plaque images, a line (1 voxel thick) was traced along the apposing membranes of the ANF and the efferent in each of the serial sections where there were synaptic specializations (see Fig. 9). Scale bar applies to all images. Thin white line on each reconstruction indicates the approximate location of the bottom of the IHCs and is roughly parallel to the sectioning plane.

As seen in the 3D reconstructions, most of these non-synaptic fibers were on the modiolar side of the IHCs (Fig. 6). The total number of ANFs in each stack, i.e. synaptic plus non-synaptic, was within the range expected in this region in a normal ear (Fig. 1B). Thus, we infer that virtually no unmyelinated terminals have disappeared, at either the 1 day or the 1 week post-exposure time.

In prior confocal studies, ~ 10% of the ribbons are “orphaned” at the 24 h post-exposure time point, i.e. they appear near the IHC membrane without any visible post-synaptic marker. Orphans are extremely rare in control ears or in exposed ears at longer post-exposure times^2^. Here, we saw a single orphan ribbon at the IHC membrane in the 1 day stack, without any ANF terminal closeby. There were no orphan ribbons in either of the control stacks or in the 1-week stack.

### C. Mitochondrial content

**Figure 7:**
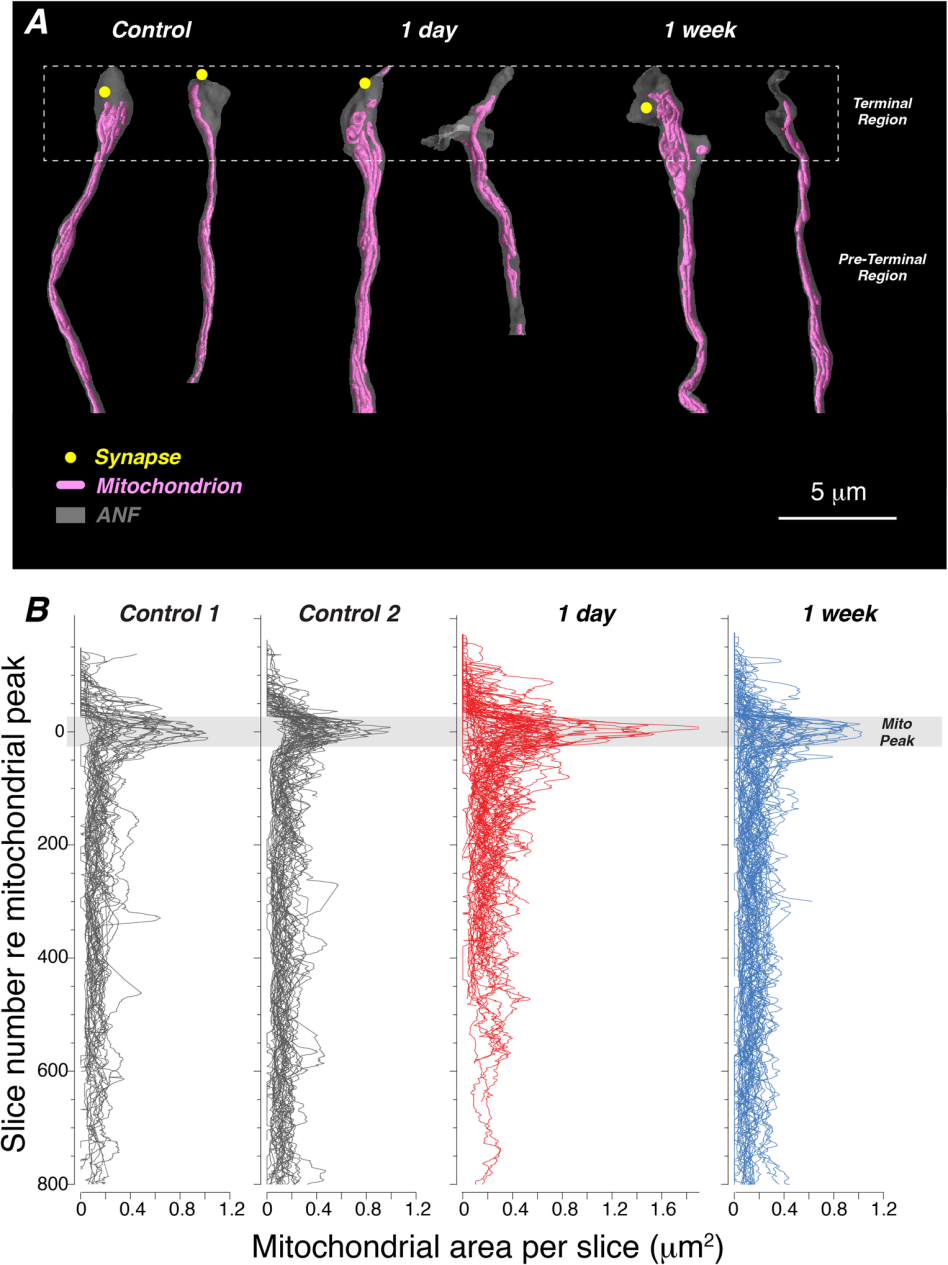
Mitochondria tend to be clustered near the synaptic region of each ANF. (**A**) Selected ANFs from each of the three exposure groups rendered to show the fiber outline and the mitochondrial content. When synapses were present, their location is indicated by the yellow circles: the synaptic ribbon is too small to be visible in renderings at this magnification. In each pair, the left-hand example is relatively rich in mitochondria, and the right-hand example is relatively poor. The terminal region is defined as the distalmost 200 slices (4 mm) of each ANF. (**B**) Most ANFs have a concentration of mitochondria near their terminal swelling. Each line in each plot shows the total mitochondrial cross-sectional area in each slice of the image stack. Slice number for each fiber is normalized to its mitochondrial peak, defined as the middle of the 50-slice segment with highest summed mitochondrial content within the terminal region. To minimize the contribution of “stretched” areas in regions where ANFs run parallel to the section plane, we rejected any ANF profile in the preterminal region in which its circularity index fell below 0.8. All ANFs from each of the four blocks are included: n = 33 from control 1, n = 41 from control 2, n = 59 from 1 day, and n = 59 from 1 week.

Mitochondrial content of ANFs is particularly relevant, because it is related to threshold sensitivity, spontaneous discharge rate and vulnerability to synaptopathy^3,13^. Exemplar 3D renderings in Fig. 7A show that, although mitochondria are present throughout much of the extent of all ANFs, there is often a clustering of mitochondria in the terminal swelling just below the extreme terminus, which is often devoid of mitochondria. This accumulation of mitochondrial area in the terminal vs. the pre-terminal regions of the fibers is illustrated in Fig. 7B, where we plot the cross-sectional area of all the mitochondria in every slice from every fiber in all four FIB-SEM stacks. The clearly non-uniform distribution of mitochondria along each fiber’s length suggest it could be useful to consider the terminal vs. pre-terminal regions separately when analyzing ANFs and the effects of noise. To that end, we divided each fiber at a point 200 slices (4 mm) from its terminus, as shown in Fig. 7A. Within the terminal region, we further define a mitochondrial-peak area as the 50-slice span with the greatest mean mitochondrial area.

**Figure 8:**
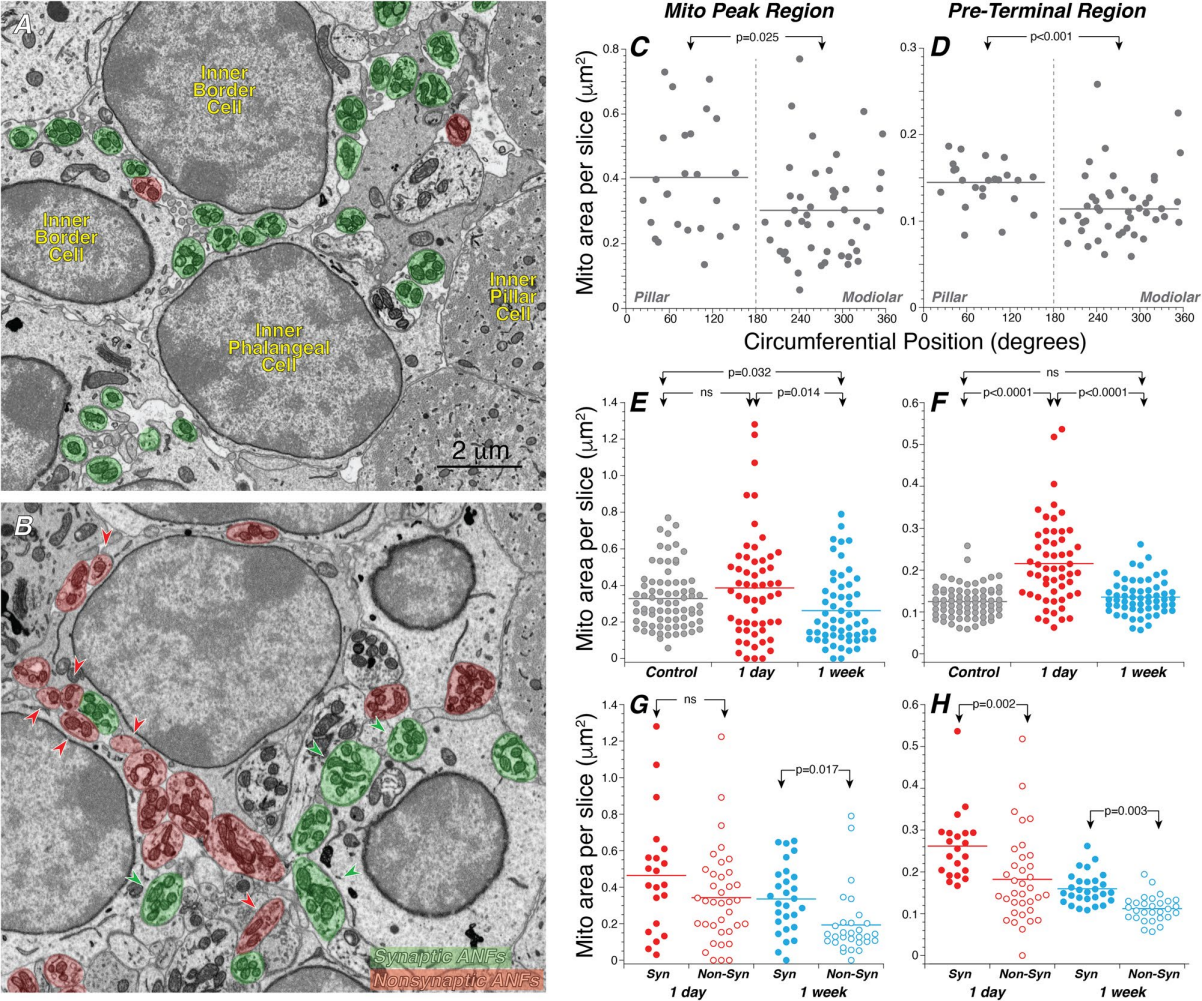
Mitochondria content is normally greater on pillar-side ANFs, and increases transiently after noise, especially in fibers that maintain synaptic connection with IHCs. (**A,B**) Representative FIB-SEM slices through the pre-terminal regions of ANFs in a control and the 1-day stack, respectively. ANFs are color coded as to whether or not they made synaptic contact with the IHC. Red arrowheads in (**B**) point to non-synaptic ANFs with relatively low mitochondrial content, and green arrowheads point to synaptic ANFs with relatively high mitochondrial content. (**C,D**) Control ANFs synapsing on the modiolar side are mitochondrion-poor compared to those on the pillar side, in both the mitochondrial-peak region (**C**) and the pre-terminal region (**D**). (**E,F**) Mitochondrial areas in the terminal (i.e. mito-peak **E**) and pre-terminal (**F**) regions seen in ANFs 1 day and 1 week after synaptopathic exposure. (**G,H**) In exposed ears, non-synapsing ANFs in both the mitochondrial peak (**G**) and pre-terminal (**H**) regions have lower mitochondrial content than synapsing fibers. Mito(chondrial)-peak and pre-terminal regions are defined as shown in Fig. 7. P values for comparisons across survival times are not indicated in (**G**,**H**). In all panels, horizontal lines on dot plots indicate the mean of each distribution. Data are from all the same fibers shown in Fig. 7.

In control ears, as expected based on prior ultrastructural studies in cat^13^, the fibers synapsing on the modiolar side of the IHC tend to be mitochondrion-poor compared to those on the pillar side, whether looking at the terminal or pre-terminal regions (Fig. 8C,D, respectively). The data in Fig. 7B also suggest that there is a transient enhancement of mitochondrial area at 1 day post exposure that largely recovers 1 week later. The further analyses of Fig. 8 show that this transient enhancement is significant only in the pre-terminal region (Fig. 8E,F). 1 week later, mitochondrial content is smaller than pre-exposure in the terminal region, but statistically indistinguishable from control in the pre-terminal region (Fig. 8E,F, respectively). Interestingly, among the ANFs in exposed ears, the non-synaptic fibers have a clear tendency towards lower mitochondrial content than the synaptic fibers, in both terminal (mito-peak) and pre-terminal regions (Fig. 8G,H, respectively).

The increases in total mitochondrial cross-sectional area could arise by swelling of individual mitochondria, or by increase in the number of mitochondrial profiles per section via elongation, division or migration^29^. The micrographs in Fig. 8A,B, suggest that the increases in mitochondrial area per slice are not due to gross swelling of individual mitochondria, indeed, the mitochondrial morphology looks quite normal in noise-exposed ANFS. To be more quantitative, we manually counted mitochondrial profiles in every 10^th^ slice of the ANFs from a control and the 1-day post-exposure cases. Results showed a 51% increase in the number of mitochondrial profiles per section (3.75 ± 0.29 vs 2.48 ± 0.13, p < 0.001) with a smaller (15%), but significant, increase in the mean size of each profile (6.22 ± 0.2 vs 5.41 ± 0.115, p = 0.002), suggesting that mitochondria are both growing in cross-sectional area and either multiplying, elongating or migrating from outside the organ of Corti to the unmyelinated terminals within the organ of Corti by 1 day post exposure.

### D. Efferent Innervation

In addition to the synaptic connection to sensory cells, ANFs in mammalian ears are innervated by terminals from the lateral olivocochlear (LOC) pathway. These efferent neurons contain several neurotransmitters including acetylcholine and dopamine^30,31^ and provide both excitatory and inhibitory feedback, respectively^32^. The dopaminergic fibers have been implicated in the protection of ANFs from noise-induced synaptopathy^33^.

**Figure 9:**
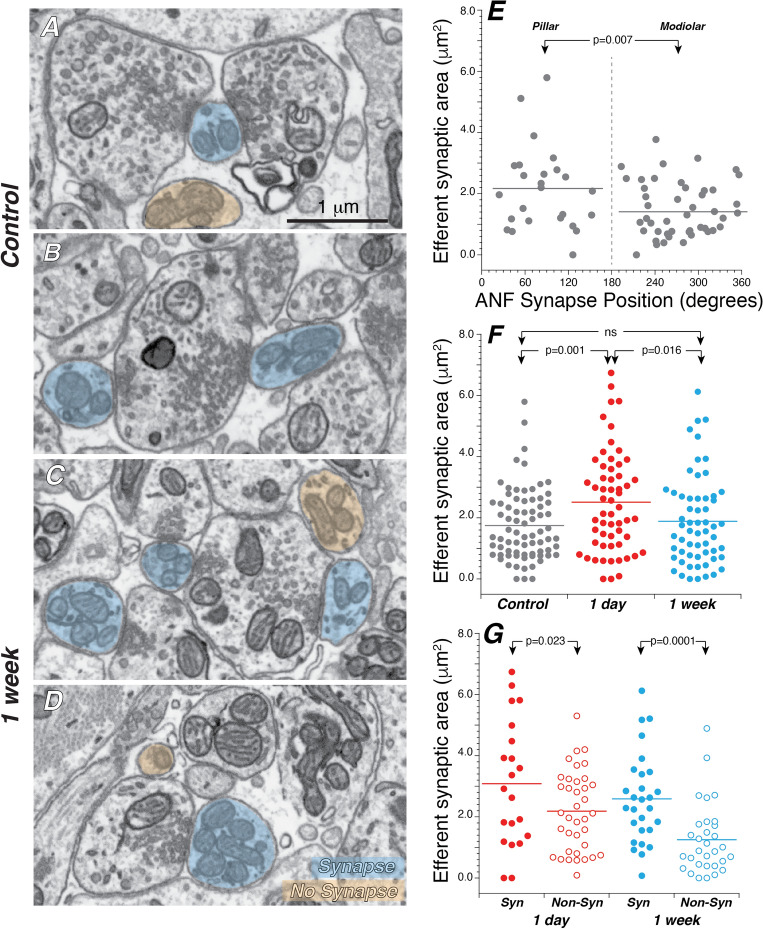
Efferent innervation is richer on pillar side ANFs and increases transiently after noise, especially in fibers that maintain synaptic contact with the IHCs. (**A–D**) FIB-SEM images from control (**A,B**) and exposed (**C,D**) ears in which some of the ANFs are highlighted in blue or orange depending on whether the contact they make with an efferent terminal is considered synaptic (blue) or not (orange). (**E**) In the control ears, modiolar-side ANFs receive less efferent innervation than pillar-side ANFs. (**F**) After exposure, the surface area of efferent synaptic contacts increases at 1 day then returns to control values at 1 week. (**G**) In exposed ears, at both 1 day and 1 week post exposure, non-synapsing ANFs have significantly less efferent innervation than ANFs that retain their synaptic connections. P values for comparisons across survival times are not indicated in (**G**). In all panels horizontal lines on dot plots indicate the mean of each distribution. Data are from all the same fibers shown in Figs. 7 and 8.

In the neuropil beneath the IHCs, LOC terminals are easily identified (Fig. 9A–D) based on their content of large clusters of synaptic vesicles^34^. LOC neurons spiral for long distances within the inner spiral bundle (Fig. 2A) and send off numerous small and large side branches to innervate many different ANFs^35^. Thus, these vesicle-filled terminals are almost always easily followed back to larger branches of highly complex neuronal structures. There are many places in the neuropil beneath the IHCs where LOC terminals come in close contact with ANFs, however, in the present study, we considered these contacts to be synaptic in nature only if the following criteria were met: (1) the membranes of the afferent and efferent fibers were in direct apposition (i.e. as close to each other as at an afferent synapse), (2) a cluster of at least five vesicles was present within two vesicle-diameters from the efferent membrane, (3) there was membrane thickening and/or intermembranous darkening at the region of apposition, and (4) criteria 1–3 were met for a minimum of five consecutive slices. Some of the synaptic and non-synaptic interactions between afferent and efferent fibers are illustrated in Fig. 9A–D.

The overall distribution of LOC synapses is well seen in the 3D reconstruction of Fig. 6 (bottom row). As expected from confocal microscopy of cochleas immunostained for cholinergic and dopaminergic markers, these synapses are concentrated in a cloud near the bases of the IHCs. In the control ears, there is a tendency for synapses on pillar-side fibers to extend farther from the IHC than on modiolar-side fibers. The superposition of elements in these projection views make it difficult to appreciate the quantitative differences in efferent innervation. These are better illustrated by summing the surface area of synaptic contact per fiber. In control ears, the modiolar-side fibers have lower areas of synaptic contact than the pillar-side fibers (Fig. 9E). In exposed ears, there is a transient increase in the area of efferent synaptic contact, that reverts back to control values at 1 week post-exposure (Fig. 9F). Interestingly, at both 1 day and 1 week post-exposure, there is significantly less efferent innervation on the non-synaptic ANFs than on the synaptic ANFs (Fig. 9G). For each of these analyses, we evaluated whether the differences in total efferent synaptic contact were due to changes in the number of contacts or the size of the existing contacts: the data suggest it is the latter. For example, the average plaque size in control vs. 1-day ANFs was 0.496  ± 0.034 μm^2^ vs. 0.666  ± 0.052 μm^2^ (a significant increase of 34%; p = 0.003). whereas the average number of plaques per ANF was 3.78 ± 0.17 vs. 3.98 ± 0.23 (an insignificant increase of 5%; p = 0.57), respectively.

## Discussion

### A. Understanding the normal innervation patterns in the mouse cochlea

Neurophysiological studies in cat suggested that ANFs can be subdivided into three functional subgroups based on their spontaneous discharge rate (SR)^36^. The evidence for this includes the observations that (1) the relation between SR and threshold is a step function with abrupt jumps at SRs of 0.5 sp/sec and 18 sp/sec, separating low- from medium- and medium- from high-SR fibers^36^, (2) the SR distribution is clearly bimodal with a null at 18 sp/s separating high-SR fibers from the low- and medium-SR group^36^, (3) the IHC synapses of low-SR fibers have multiple pre-synaptic ribbons, whereas those of medium- and high-SR fibers do not^37^ and (4) the central projections of low-SR fibers innervate the small-cell cap of the cochlear nucleus, whereas medium- and high-SR fibers do not^38^.

In mouse, ANFs show the same basic relation between threshold and SR, i.e. the higher the SR the lower the threshold, however the relation is a continuum rather than a step function, and the SR distribution is not obviously bimodal^39^. Nevertheless, cluster analysis of gene expression in single SGNs in mouse also suggest three distinct subgroups^40^. Furthermore, morphological analysis suggested that types 1a, b and c SGNs correspond to high-, medium- and low-SR groups, respectively, because 1b and 1c gene-expression subclasses exclusively innervate the modiolar side of the IHC^40^, as was observed for medium- and low-SR physiological classes in cat^11^.

In the present study, we show that fibers innervating the modiolar side of the IHC are mitochondrion-poor with respect to those on pillar side (Fig. 8C,D), as seen in prior ultrastructural studies in cat^13^. This makes functional sense, given that mitochondria are ATP generators and that fibers with lower rates of spontaneous spike activity must consume less energy. The functional significance of other modiolar-pillar differences is not so clear. For example, prior ultrastructural studies in cat^14^ and mouse^12,28^ showed that pre-synaptic ribbons on the modiolar side are larger than those on the pillar side. The same trend was seen here, though the spatial difference only reached statistical significance in the 1-day post-exposure case (Fig. 4). A prior ultrastructural study in mouse suggested that the normal ribbon gradient is only apparent when the modiolar-pillar distinction is defined based on each IHC in isolation, rather than by a single “average” plane for all the IHCs in the region of interest^28^. This difference in definition can dramatically alter ANF modiolar-pillar classification, because the position of the IHC synaptic poles in mouse are often staggered, with one tilting towards the modiolus and the next tilting towards the pillars^41^. However, the present analyses of modiolar-pillar ribbon gradients reached the same conclusion regardless of which definition was used, and our prior confocal analyses saw significant gradients even when using a “single-plane” to separate modiolar from pillar sides of a z-stack containing 10–12 adjacent IHCs^17,41^ .

Another curious spatial difference in prior studies is that synaptic ribbons associated with low-SR/modiolar ANFs are more often present in pairs^12,37^. Here, we saw only two double ribbons out of 71 synapses in control ears (3%), one each of the pillar and modiolar side vs. 27%^12^ and 11%^28^ in prior serial-section ultrastructural studies in mouse. One of the prior studies^12^ also suggested a higher fraction of branching ANFs (37%) than seen here (3%). These quantitative differences could be due to strain differences (CBA/Ca^12^ vs. C57/Bl/6J^28^ vs. CBA/CaJ here), sex difference (females^12^ vs. males here) or cochlear region differences (mid-cochlear^12,28^ vs. mid-basal here). The latter may be the most likely, since prior study suggested that branching was more common towards the apex of the cochlea^14^.

Prior ultrastructural studies in both mouse^12^ and cat^34^ found significantly more synaptic contacts from olivocochlear efferents onto modiolar-side ANF terminals than pillar-side terminals. Here, in contrast, we saw the opposite trend (Fig. 9E). In addition to the inter-study differences cited above, the present study used a different metric, i.e. the total surface area of the putative synaptic interfaces rather than simply the number of such contacts, as was done in both prior studies. However, using the latter metric on the present dataset does not change the result or resolve the differences. In the cat study, synaptic contacts were easily distinguishable from simple membrane apposition, because of the electron-dense spikes extending from the pre-synaptic membrane towards the vesicle clusters in the efferent terminals^34^. Here we saw no such pre-synaptic spikes and defined efferent synapses based on apposition, vesicle clusters and a more diffuse type of electron density at the point of contact (Fig. 9A–D). The prior mouse study^12^, which had a lower resolution in x and y (11 vs 5 nm per pixel), and used only vesicle clustering and membrane apposition as a criterion for an efferent synapse, likely classified more cell-to-cell contacts as synaptic in nature than we did here, and this could contribute to the difference in results *re* the modiolar-pillar balance of efferent synapses.

### B. Clarifying the dynamics of noise-induced synaptopathy

It has long been known that overexposure to noise can cause hair cell death^42^ and/or irreversible damage to their stereocilia^43^, either of which results in permanent threshold elevation^44^. In the 1980’s, a number of ultrastructural studies reported dramatic swelling and membrane rupture of ANF terminals in the IHC area, apposed to normal looking pre-synaptic ribbons, when cochleas were examined within 24 h post-exposure^20,21^. Because this pathology was absent in noise-exposed ears examined at later time points, and could be elicited by exposures for which the threshold shifts were also ultimately reversible, it was assumed that this noise-induced neuropathy was fully reversible^45^. However, these ultrastructural studies used a random-section approach. Since none reconstructed the entire neuropil, no ANF counts were made, and ANF contacts that had lost synaptic specialization would not have been appreciated, given that even normal ANFs have long regions of close apposition to IHCs outside the active zone of pre- and post-synaptic specialization.

Interest in cochlear synaptopathy was revived when confocal studies of immunostained pre-synaptic ribbons and post-synaptic glutamate receptor patches in large numbers of IHCs showed that even exposures causing only temporary threshold elevations could cause an immediate, irreversible loss of synapses followed by a very slow degeneration of a comparable number of SGNs that required years to fully manifest^1^. Evidence that noise-induced synaptopathy is a type of glutamate excitotoxicity includes the observations that (1) ANF terminal swelling can be induced by cochlear perfusion of glutamate agonists^46^, (2) noise-induced ANF swelling can be reduced by perfusion of glutamate antagonists^46^, and (3) noise-induced synaptic loss can be reduced by genetic deletion of the gene for glutamate transport into synaptic vesicles^47^ or specific glutamate receptor blockade^8^.

Prior confocal study of this synaptopathic mouse model has shown that ribbon counts decrease immediately post-exposure by 50% at the 32 kHz region^2,5,23^. Here, we show the same fractional decrease in ribbon counts at 32 kHz, both 1 day and 1 week post exposure (Fig. 1), but reveal that ANFs at roughly half of these “missing” synapses remain intact, with an extensive region of IHC contact, and surprisingly normal morphology, including supranormal mitochondrial content (Fig. 8) and an abnormally rich efferent innervation (Fig. 9). The other half of the missing synapses reflect ANF terminals that have partially retracted from the IHC membrane, although most of these remain close (~ 2 mm) to the IHCs. The latter group may correspond to the subset of terminals which early ultrastructural studies saw as grossly swollen with ruptured membranes when viewed immediately post-exposure^20,21,45^.

It is tempting to speculate that the non-synaptic fibers are predominately low-SR/type 1c and medium-SR/type 1b ANFs, with the partially retracted fibers corresponding to the former. Indeed, at both 1 day and 1 week post exposure, most of the non-synaptic fibers contacted (or were headed towards) the modiolar side of the IHCs, suggesting that they had generally lower mitochondrial content pre-exposure as well as higher thresholds and lower SR. They clearly had lower mitochondrial content post-exposure (Fig. 8G,H). It is likely that a lower mitochondrial content predisposes ANFs to glutamate excitotoxicity, given that Ca^++^ overload is a major contributor, and mitochondria are an important source of intracellular Ca^++^ buffering^48^. The transient proliferation of mitochondria in the synaptic fibers at 1 day post-exposure could reflect an adaptive response that supports their resistance to the overstimulation. The transient increase in efferent innervation in the remaining synaptic fibers could also contribute to this resistance, given the hypothesized role of these olivocochlear efferents in minimization of excitotoxicity^33^ and thus in minimizing synaptopathy.

Permanent synaptopathy would not cause permanent threshold shift if the neural loss were selective for (high-threshold) fibers with low- and medium-SR. However, it would be expected to cause problems with stimulus coding in a noisy environment, since the high-threshold fiber are less affected by moderate levels of noise, simply because their thresholds are higher^6^. Indeed, a prior neurophysiological study of single ANFs in synaptopathic guinea pigs found an altered SR distribution consistent with selective loss of low- and medium-SR fibers^3^. Although an ANF study in synaptopathic mice did not see SR alterations consistent with this selective loss^5^, the modiolar predilection for synaptopathy observed here is consistent with selective damage to high-threshold fibers.

In this mouse model of noise-induced synaptopathy, single-fiber neurophysiology^5^ showed an unexpected decrease in the mean temporal jitter of the onset responses to short tone bursts of the type used to elicit auditory evoked potentials like the auditory brainstem response (ABR) or the compound action potential (CAP). This enhancement of the responses of the surviving fibers would also contribute to the insensitivity of ABR or CAP thresholds to the noise-induced synaptopathy. It is tempting to speculate that this response enhancement might arise from ribbon multiplication and hypertrophy at the surviving synapses (Figs. 3, 4B). All other things being equal, the associated increase in the number of synaptic vesicles tethered in close proximity to the pre-synaptic membrane at surviving high-SR synapses could increase the size of the post-synaptic EPSCs and thereby increase the onset synchrony, but additional morphological data are needed to determine if these differences are really significant.

### C. Relevance to regenerative strategies

The partial de-afferentation of surviving IHCs has also been documented in aging mice^49^ and in aging humans^50^, where it is also exacerbated by a history of noise exposure^51^. In the human cochlea, the synaptic ribbons and myelinated peripheral axons disappear, while many of the cell bodies of the ANFs (as well as their central axons) remain intact. Thus, synaptopathy is important clinically, and efforts at re-establishing ANF synapses have relevance to human hearing impairment.

Cochlear delivery of an endogenous neurotrophin, NT3, can rescue noise-induced synaptopathy if carried out within 24 h, but not at 1 week post exposure^23^. This qualitative effect of a one-week difference in trauma-treatment interval is what inspired the choice of survival times in the present study. Without the ultrastructural insights from the present study, it was assumed that the ANF peripheral terminals ruptured immediately post exposure and that the preterminal unmyelinated dendrites then began to retract towards the first node of Ranvier located just outside of the sensory epithelium. It was attractive to hypothesize that the 1 day vs. 1 week differential in the effectiveness of NT3 therapy reflected this progressive retraction. Here, we show that this is not the explanation. In fact, most of the synaptopathic ANF terminals remain in contact or very close contact with the IHCs, and there is little difference, in this regard, between the 1 day and the 1-week post-exposure times (Fig. 6). The observed changes in mitochondrial content and efferent innervation between the two post exposure times could reflect underlying alterations in metabolic state that might relate to the difference in therapeutic effectiveness, but provide little insight into how to overcome them. The most important insight from the present results is that, contrary to the inferences drawn from in vitro studies of glutamate excitotoxicity^22^, the ANF dendrites do not all rupture and retract towards the point of myelination in the habenula. The surprisingly long post-exposure survival of the unmyelinated dendrites in direct contact with IHCs suggests that the emphasis in therapeutic strategies should shift from evoking neurite extension to eliciting synaptogenesis.

## Data Availability

The data that support the findings of this study are available from the corresponding author upon reasonable request.
